# First checklist of the fruit flies of Morocco, including new records (Diptera, Tephritidae)

**DOI:** 10.3897/zookeys.702.13368

**Published:** 2017-09-26

**Authors:** Younes El Harym, Boutaïna Belqat

**Affiliations:** 1 Department of Biology, Faculty of Sciences, University Abdelmalek Essaâdi, Tétouan

**Keywords:** Anti Atlas, Beni Snassen, High Atlas, Middle Atlas, North Africa, Rif, Sahara

## Abstract

The first checklist of the Tephritidae of Morocco, containing 59 species, is presented here. Out of 38 species collected during the present project, three (*Campiglossa martii* (Becker, 1908), *Tephritis divisa* (Rondani, 1871), and Terellia sp. near longicauda) present new records for North Africa, and ten (*Carpomya incompleta* (Becker, 1903), *Chaetorellia conjuncta* (Becker, 1913), *Chetostoma curvinerve* Rondani, 1856, *Dacus frontalis* (Becker, 1922), *D. longistylus* (Wiedemann, 1830), *Dioxyna sororcula* (Wiedemann, 1830), *Ensina sonchi* (Linnaeus, 1767), *Myopites inulaedyssentericae* Blot, 1827, *M. stylatus* Fabricius, 1794, and *Tephritis vespertina* (Loew, 1844)) are new for Morocco.

## Introduction

Tephritidae is one of the largest families of the acalyptrate Diptera, and more than 4300 valid species are known in the world (Norrbom 2004). Prior to this study, the Tephritid fauna of Morocco has not been the subject of focused research. Only few studies from North Africa were devoted to the fruit flies (e.g., Tunisia, Arambourg and Soria 1961; Algeria, Hering 1937; Morocco, Algeria and Tunisia, Heřman and Dirlbek 2006). Some new species were described from Algeria (Dirlbek and Dirlbekova 1976; Hendel 1927; Hering 1938; Séguy 1934a) and Morocco (Becker and Stein 1913; Séguy 1930, 1941).

The Tephritidae of Morocco have been also published upon among some Diptera studies (Wiedemann 1824; Becker and Stein 1913; Séguy (1930, 1934b, 1941, 1949, 1953). Faunal records from all relevant publications have been registered in the Catalogue of the Tephritidae of the World (Norrhbom et al. 1999). A total of 46 species have been previously recorded from Morocco. New findings increase the number of fruit flies known from Morocco to 59.

The material reported in the present article was mostly collected in Morocco between 2013–2016 in 99 field expeditions over mountainous areas (Rif, Beni Snassen (eastern Morocco), Middle Atlas, Anti Atlas) and the arid area of Morocco (Sahara). Thirty-eight species of Tephritidae were identified from this material, of which ten species are new to Morocco, and three species are new to North Africa. One of the main achievements of this project is the first checklist of Tephritidae from Morocco, containing 59 species, which is presented here.

## Materials and methods

### Collecting methods

A total of 924 specimens of Tephritidae were collected by sweeping nets and malaise traps, or reared from flower heads or fruits of plants, examined, and preserved in 70% ethanol. When terminalia were necessary to confirm species identity, they were prepared by boiling the abdomen in 10% KOH for 20 minutes at 95°C and preserved in glycerine. Species were recognised according to the identification keys of Hendel (1927), Freidberg and Kugler (1989), White and Elson-Harris (1992), Merz (1994), Korneyev and White (1992, 2000). The systematic classification is based on White and Elson-Harris (1992), Norrbom et al. (1999), White et al. (2000) and White and Goodger (2009).

**Table 1.** Species (in alphabetical order) of Tephritidae known from North Africa.

Species	Morocco	Algeria	Tunisia	Libya	Egypt
*Acanthiophilus helianthi* (Rossi, 1794)	X*	X	X		
*Aciura coryli* Rossi, 1794	X				X
*Bactrocera cucurbitae* (Coquillett, 1899)					X
*Bactrocera oleae* Rossi, 1790	X**				X
*Bactrocera zonata* Saunders, 1842				X	
*Campiglossa hofferi* (Dirlbek & Dirlbekova, 1976)		X			
*Campiglossa martii* (Becker, 1908)	X****				
*Campiglossa producta* Loew, 1844	X*	X	X		
*Capitites ramulosa* (Loew, 1844)	X	X			X
*Capparimyia savastani* (Martelli, 1911)	X	X	X	X	X
*Carpomya incompleta* (Becker, 1903)	X***				X
*Carpomya pardalina* (Bigot, 1891)					X
*Ceratitis capitata* (Wiedemann, 1824)	X*	X	X	X	X
*Chaetorellia conjuncta* (Becker, 1913)	X***				X
*Chaetorellia hestia* (Hering, 1937)	X**	X			
*Chaetorellia succinea* Costa, 1844	X				X
*Chaetostomella cylindrica* Robineau-Desvoidy, 1830	X	X	X		
*Chetostoma curvinerve* Rondani, 1856	X***	X?	X?	X?	X?
*Dacus annulatus* (Becker, 1903)					X
*Dacus ciliatus* (Loew, 1862)					X
*Dacus frontalis* (Becker, 1922)	X***	X		X	X
*Dacus longistylus* (Wiedemann, 1830)	X***			X	X
*Dacus semisphaereus* (Becker, 1903)					X
*Dacus sexmaculatus* (Walker, 1871)					X
*Desmella conyzae* (Frauenfeld, 1857)					X
*Desmella rostellata* (Séguy, 1941)	X				
*Dioxyna bidentis* (Robineau-Desvoidy, 1830)	X ?	X?	X?	X?	X?
*Dioxyna sororcula* (Wiedemann, 1830)	X***	X	X		
*Ensina sonchi* (Linnaeus, 1830)	X***	X			
*Euaresta bullans* (Wiedemann, 1830)	X*	X	X		
*Euarestella iphionae* (Efflatoun, 1924)					X
*Euarestella kugleri* (Freidberg, 1974)					X
*Euarestella pninae* (Freidberg, 1981)					X
*Euleia heraclei* (Linnaeus, 1758)	X**	X			
*Euleia marmorea* (Fabricius, 1805)	X				
*Goniurellia lacerata* (Becker, 1913)					X
*Goniurellia longicauda* (Freidberg, 1980)	X**	X	X	X	X
*Goniurellia persignata* (Freidberg, 1980)	X*				X
*Goniurellia spinifera* (Freidberg, 1980)					X
*Hyalotephritis planiscutellata* (Becker, 1903)					X
*Hypenidium graecum* (Loew, 1862)	X				
*Katonaia aida* (Hering, 1938)					X
*Metasphenisca gracilipes* (Loew, 1862)					X
*Metasphenisca haematopoda* (Bezzi, 1924)					X
*Metasphenisca negeviana* (Freidberg, 1974)					X
*Myopites apicatus* (Freidberg, 1979)	X				
*Myopites boghariensis* (Séguy, 1934)		X			
*Myopites cypriacus* Hering, 1938	X				
*Myopites inulaedyssentericae* Blot, 1827	X***	X			
*Myopites stylatus* Fabricius, 1794	X***	X	X?	X?	X?
*Myopites variofasciatus* (Becker, 1903)					X
*Neoceratitis efflatouni* (Hendel, 1931)					X
*Notomma mutilum* Bezzi, 1923					X
*Oedaspis daphnea* (Séguy, 1930)	X				
*Oedaspis farinosa* (Hendel, 1927)		X			
*Oedaspis fissa* Loew, 1862		X			
*Oedaspis multifasciata* (Hering, 1937)	X	X			
*Oedaspis trotteriana* (Bezzi, 1913)	X	X		X	X
*Oedaspis villeneuvei* (Bezzi, 1913)		X		X	X
*Oxyaciura tibialis* Robineau-Desvoidy 1830	X*				
*Oxyna superflava* (Freidberg, 1974)	X				X
*Paradesis augur* (Frauenfeld, 1857)		X	X		X
*Paraspheniscus debskii* (Efflatoun, 1924)					X
*Schistopterum moebiusi* (Becker, 1903)					X
*Spathulina acroleuca* (Schiner, 1868)					X
*Spathulina sicula* (Rondani, 1856)	X*		X		
*Sphaeniscus filiolus* (Loew, 1869)	X**				X
*Sphenella marginata* (Fallen, 1814)	X*				X
*Tephritis bimaculata* (Freidberg, 1981)					X
*Tephritis dioscurea* Loew, 1856	X				
*Tephritis divisa* (Rondani, 1871)	X****				
*Tephritis formosa* (Loew, 1844)	X**				
*Tephritis jabeliae* (Freidberg, 1981)					X
*Tephritis leontodontis* De Géer, 1776	X	X?	X?	X?	X?
*Tephritis matricariae* (Loew, 1844)	X*				X
*Tephritis nigricauda* Loew 1856	X**	X			X
*Tephritis postica* (Loew, 1844)	X**	X	X		
*Tephritis praecox* (Loew, 1844)	X**	X			
*Tephritis pulchra* Loew, 1844	X	X			
*Tephritis simplex* (Loew, 1844)	X	X	X		
*Tephritis stictica* Loew, 1862	X				
*Tephritis spreta* (Loew, 1861)					X
*Tephritis theryi* (Séguy, 1930)	X				
*Tephritis truncata* (Loew, 1844)			X		
*Tephritis vespertina* (Loew, 1844)	X***		X		
*Tephritomyia lauta* (Loew, 1869)	X**		X		X
*Terellia colon* Meigen, 1826	X	?	X	?	?
*Terellia fuscicornis* (Loew, 1844)	X	X?	X?	X?	X?
*Terellia longicauda* Meigen, 1838	X				
*Terellia luteola* (Wiedemann, 1830)			X		X
*Terellia oasis* (Hering, 1938)		X			
*Terellia serratulae* (Linnaeus, 1758)	X*	X	X		
Terellia sp. near longicauda	X****				
*Terellia vectensis* Collin, 1937		X			
*Terellia virens* (Loew, 1846)	X**		X		
*Trupanea amoena* (Frauenfeld, 1857)	X**		X		
*Trupanea guimari* (Becker, 1908)	X**	X			
*Trupanea pseudoamoena* (Freidberg, 1974)					X
*Trupanea repleta* (Bezzi, 1918)					X
*Trupanea stellata* (Fuesslin, 1775)	X**		X		
*Urelliosoma desertorum* (Efflatoun, 1924)					X
*Urelliosoma pulcherrimum* (Efflatoun, 1924)		X	X		X
*Urophora calcitrapae* (White & Korneyev, 1989)					X
*Urophora mauritanica* (Macquart, 1851)	X**	X		X	
*Urophora pauperata* Zaitzev, 1945					X
*Urophora phaeocera* (Hering, 1961)					X
*Urophora quadrifasciata* (Meigen, 1826)	X	X	X		
*Urophora quadrifasciata algerica* (Hering, 1941)		X	X		
*Urophora solstitialis* Linnaeus 1758	X	X	X		

X****: new species for North Africa;

X***: new species for Morocco;

X**: new species for one or more of the geographic regions of Morocco;

X*: species known from Morocco and collected by the authors;

?: species considered, in the literature, present in North Africa without specifying country.

**Map 1.**
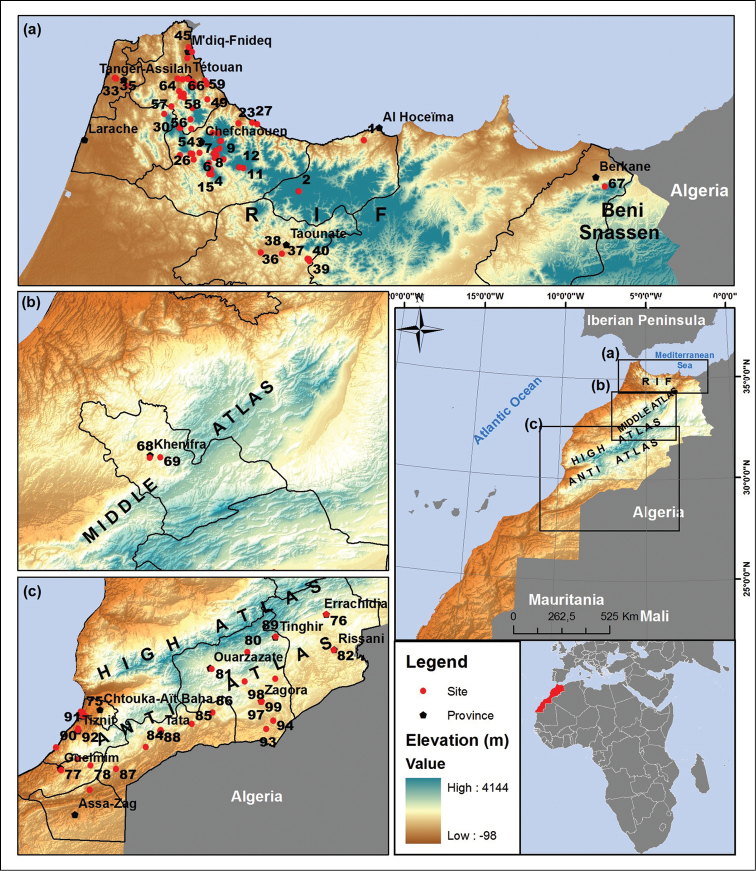
Map showing all collecting sites in Morocco.

A list of 99 sampling sites, with coordinates and altitudes, is given in Table 2, and the locations of the sites are shown in Map 1, elaborated using the logiciel GisArc (Geographic Information System, version 9.3). All the specimens are deposited in the collection of Diptera of the Department of Biology, Faculty of Sciences, University Abdelmalek Essaâdi, Tétouan.

**Table 2.** Sampling sites (in alphabetical order) harboring the species collected in Morocco with localities, geographical coordinates, and altitudes.

**Province**	**Station**	**Locality**	**Altitude (m)**	**Geographical coordinates**
**Rif**				
Al Hoceïma	1. Affluent Tarmast	Parc National d’Al Hoceïma	168	N35°10.666', W004°03.088'
	2. Oued Azila	Azila, Jbel Tidghine	1601	N34°52.028', W004°32.609'
Chefchaouen	3. Aïn Afersiw	Mezine	746	N35°05.945', W005°20.439'
	4. Aïn El Ma Bared	Bouzthate	1267	N35°00.333', W005°12.105'
	5. Aïn El Malâab	Parc National Talassemtane	1278	N35°05.509', W005°09.443'
	6. Aïn El Maounzil	Parc National Talassemtane	1106	N35°04.577', W005°10.406'
	7. Aïn Tiouila	Parc National Talassemtane	1502	N35°07.194', W005°09.978'
	8. Bab El Karne	Tamakoute	1248	N34°58.510', W005°11.838'
	9. Cascade Chrafate	Chrafate	820	N35°03.997', W005°06.434'
	10. Daya Afrate	Tanaqoub	600	N35°05.634', W005°26.028'
	11. Daya El Ânassar	Bab Berred	1183	N35°00.788', W004°57.419'
	12. Daya El Birdiyel	Ânasar	1300	N35°01.089', W004°59.477'
	13. Douar Abou Boubnar (Marabout Sidi Gile)	Parc National Talassemtane	1247	N35°10.812', W005°07.500'
Chefchaouen	14. Douar Ouslaf	Parc National Talassemtane	625	N35°13.787', W005°11.376'
	15. Douar Tamakoute	Tamakoute	1089	N34°58.609', W005°12.743'
	16. Maison forestière	Parc National Talassemtane	1674	N35°08.076', W005°08.262'
	17. Mizoghar	El Khizana	1005	N35°02.725', W005°12.969'
	18. Oued Abou Bnar	Parc National Talassemtane	1260	N35°10.854', W005°07.840'
	19. Oued Derdara	Derdara	400	N35°06.484', W005°17.147'
	20. Oued El Kanar	Stehate	50	N35°17.233', W004°59.639'
	21. Oued El Kelâa	Akchour	510	N35°13.514', W005°08.553'
	22. Oued Jbara	Tanaqoub	600	N35°05.634', W005°26.028'
	23. Oued Jnane Niche	Jnane Niche	36	N35°17.019', W004°51.233'
	24. Oued Majjou	Village Majjou	799	N35°06.186', W005°10.935'
	25. Oued Mezine	Mezine	778	N35°06.104', W005°21.177'
	26. Oued Sidi Ben Sâada	Derdara	220	N35°03.921', W005°19.895'
	27. Oued Sidi Yahya Aârab	Sidi Yahya Aârab	62	N35°17.545', W004°53.503'
Larache	28. Affluent Oued Amsemlil	Jbel Bouhachem	1130	N35°15.657', W005°26.159'
	29. Daya Mtahen	Jbel Bouhachem	966	N35°16.195', W005°26.158'
	30. Daya Tazia	Route Moulay Abdessalam	721	N35°20.814', W005°33.139'
	31. Oued Tkaraâ	Jbel Bouhachem	959	N35°16.063', W005°25.829'
M’diq-Fnideq	32. Ksar Rimal	Kabila	11	N35°43.806', W005°20.509'
Tanger-Assilah	33. Daya Aïn Jdioui	Aïn Jdioui	5	N35°34.074', W005°55.499'
	34. Daya El Hajjami	Barrage 9 Avril	21	N35°31.306', W005°50.263'
	35. Oued Aïn Jdioui (Touaret)	Aïn Jdioui	2	N35°33.768', W005°55.002'
Taounate	36. Aïn Boharroch	Aïn Aïcha	234	N34°29.049', W004°40.442'
	37. Dhar Sbagh Mâasra	Khemis Hoara	396	N34°26.977', W004°28.972'
	38. El Hajria	Sahel Botaher	184	N34°29.496', W004°49.886'
	39. Koudia El Aouinate	Douar Kda	403	N34°26.170', W004°28.167'
	40. Lâazaba	Khemis Hoara	357	N34°26.820', W004°28.408'
Tétouan	41. Aïn El Âakba Larbaâ	Mokdassene	95	N35°31.328', W005°19.012'
	42. Barrage Nakhla	Zinat	240	N35°26.954', W005°24.326'
	43. Barrage Smir	Bouzaghlale (M’diq)	20	N35°41.491', W005°22.673'
	44. Daya Amsemlil	Jbel Bouhachem	1059	N35°15.596', W005°25.917'
	45. Daya Jbel Zemzem	Jbel Zemzem	216	N35°45.457', W005°22.189'
	46. Douar Dacheryène	Dacheryène	132	N35°33.863', W005°27.162'
	47. Douar Kitane	Kitane	52	N35°32.412', W005°20.393'
	48. Douar Taghbaloute	Larbaa Beni Hassen	379	N35°15.323', W005°20.887'
	49. Douar Tizga	Amsa	516	N35°26.237', W005°13.694'
	50. El Haouta	Kitane	52	N35°32.230', W005°20.166'
	51. El Malâab	Kitane	75	N35°32.348', W005°20.290'
	52. Jumb Kitane	Kitane	38	N35°32.759', W005°20.420'
	53. Oued Achekrade	Douar Aouzighen	642	N35°22.931', W005°20.364'
	54. Oued Al Mizzine	Mezine	790	N35°06.143', W005°21.272'
	55. Oued Amsa	Amsa	11	N35°31.926', W005°13.747'
Tétouan	56. Oued Boumarouil	Larbaa Beni Hassen	551	N35°18.733', W005°21.271'
	57. Oued El Hamma	Beni Ydder	200	N35°23.532', W005°30.051'
	58. Oued El Kebir	Route Vers Beni Yedder	89	N35°27.351', W005°25.796'
	59. Oued Halila	Azla	3	N35°33.137', W005°14.678'
	60. Oued Maâza (Âachira)	Kitane	15	N35°32.596', W005°20.350'
	61. Oued Maâza (Tarik El Ouasâa)	Kitane	39	N35°32.237', W005°19.891'
	62. Oued Martil	Mhannech 2	9	N35°33.693', W005°22.510'
	63. Oued Martil (Tamouda)	Tamouda	8	N35°33.627', W005°24.799'
	64. Oued Sahel	Ben Karrich	40	N35°29.238', W005°26.352'
	65. Oued Zarka	Zarka	128	N35°31.211', W005°20.477'
	66. Oued Zinat	Zinat	190	N35°28.207', W005°24.224'
**Beni Snassen (Eastern Morocco)**				
Berkane	67. Oued Beni Ouaklane	Beni Snassen	630	N34°51.581', W002°15.180'
**Middle Atlas**				
Khénifra	68. Oued Oum Er Rbia	Farra	826	N32°55.358', W005°40.390'
	69. Sensla	Arougo	1111	N32°55.387', W005°34.081'
**Anti Atlas**				
Assa zag	70. Oued Assa	Assa	306	N28°36.507', W009°25.800'
Chtouka-Aït Baha	71. Avant Sidi Binzarne	Massa	10	N30°03.144', W009°39.067'
	72. Centre Sidi Ouassay	Massa	41	N30°03.387', W009°41.249'
	73. Douar Sidi Abou	Massa	55	N29°59.322', W009°35.627'
	74. Environs Massa	Massa	24	N29°59.353', W009°38.708'
	75. Oued Massa (Pont Aghbalou)	Massa	2	N30°02.007', W009°38.768'
Errachidia	76. Oued Ziz (Pont Errachidia)	Oued Ziz	1026	N31°56.253', W004°25.455'
Guelmim	77. Msidira	El Filaha	257	N28°56.865', W010°02.979'
	78. Oued Sayad	Taghjijt	568	N29°03.784', W009°26.027'
	79. Oued Tisla	Bouizakarne	613	N29°09.908', W009°43.280'
Ouarzazate	80. Douar Zaouia	Kelâa M’gouna	1238	N31°14.275', W006°08.169'
	81. Oued Ouarzazate	Ouarzazate	1135	N30°54.917', W006°54.187'
Rissani	82. Ksibat Elhdeb	Rissani	770	N31°16.494', W004°16.310'
Tata	83. Douar Tighrimt	Tighrimt	706	N29°45.742', W007°58.521'
	84. Douar Zaouiet	Akka	554	N29°26.485', W008°16.535'
	85. Imzine	Tissint	566	N29°53.415', W007°18.928'
	86. Oued Foum Ziguid (Douar Ouaiftoute)	Foum Ziguid	665	N30°06.786', W006°52.639'
	87. Oued Tamanarne	Foum El Hisn	480	N29°00.941', W008°53.588'
	88. Oued Tata	Tata	277	N29°45.348', W007°58.690'
Tinghir	89. Oued Tinghir	Douar Ihertane	1280	N31°31.366', W005°31.677'
Tiznit	90. Airport Sidi Ifni	Sidi Ifni	51	N29°22.654', W010°10.711'
	91. Atbane	Tiznit	191	N29°44.388', W009°43.740'
	92. Route Bab El Khemis	Tiznit	225	N29°42.647', W009°43.702'
Zagora	93. Aït Aissa O Brahim	Mhamid El Ghizlane	556	N29°48.948', W005°43.676'
	94. Douar Rggaga	Tagonite	614	N29°57.874', W005°34.532'
	95. Isdaoun	Tazzarine	852	N30°44.748', W005°32.194'
	96. Jnane Makadir	Zagora	726	N30°19.209', W005°50.498'
	97. Kasbah Asma	Amzrou	718	N30°19.015', W005°50.016'
	98. Oued Drâa (Ikhf Mezrou)	Zagora	872	N30°41.466', W006°11.405'
	99. Oued Drâa (Tahtah)	Zagora	718	N30°19.665', W005°49.813'

The following checklist summarizes the species inventory presently known from Morocco, and their worldwide distribution.

## Results

### Faunistic records

#### Family TEPHRITIDAE (Newman, 1834)

##### Subfamily DACINAE (Loew, 1862)

###### Tribe CERATITIDINI Bezzi, 1910

####### Genus *CAPPARIMYIA* Bezzi, 1920

######## Capparimyia savastani

Taxon classificationAnimaliaDipteraTephritidae

Martelli, 1911

######### Literature records.

Morocco, Anti Atlas: Tiznit (Séguy 1953).

######### World distribution.

Algeria, Tunisia, Libya, Egypt, Italy, Greece, Malta, Israel, Oman, France, Yemen and Pakistan (Freidberg and Kugler 1989, Norrbom et al. 1999, Donati and Belcari 2003, De Meyer and Freidberg 2005, Miranda et al. 2008, Papachristos et al. 2009).

####### Genus *CERATITIS* MacLeay, 1829

######## Ceratitis capitata

Taxon classificationAnimaliaDipteraTephritidae

(Wiedemann, 1824)

######### Literature records.

Morocco, Rif: Tangier (Becker and Stein 1913), High Atlas (Séguy 1930), Ouadj-Ouli-Mohamed, env. Settat, Insgane (De Meyer 2000).

######### New records.

Morocco, Rif: Douar Kitane, 4♂♂, 4♀♀, 23-IX-2014- 4♂♂, 24-IX-2014- 4♂♂, 3♀♀, 25-IX-2014- 1♂, 2♀♀, 27-IX-2014- 1♂, 3♀♀, 02-X-2014- 4♂♂, 1♀, 27-VII-2015 (reared); El Haouta, 1♂, 1♀, 20-IX-2014 (net sweeping)- 5♂♂, 11♀♀, 02-X-2014 (reared); Jumb Kitane, 4♂♂, 7♀♀, 30-VI-2015. Middle Atlas: Sensla, 1♀, 14-VII-2016 (net sweeping). Anti Atlas: Environs Massa, 1♀, 11-V-2015; Oued Massa (Pont Aghbalou), 2♂♂, 12-V-2015; Douar Sidi Abou, 23♂♂, 34♀♀, 13-V-2015; Douar Tighrimt, 1♂, 29-V-2015; Douar Zaouia, 1♂, 11-VI-2015 (net sweeping).

######### Host plants.

**Figure 1.**
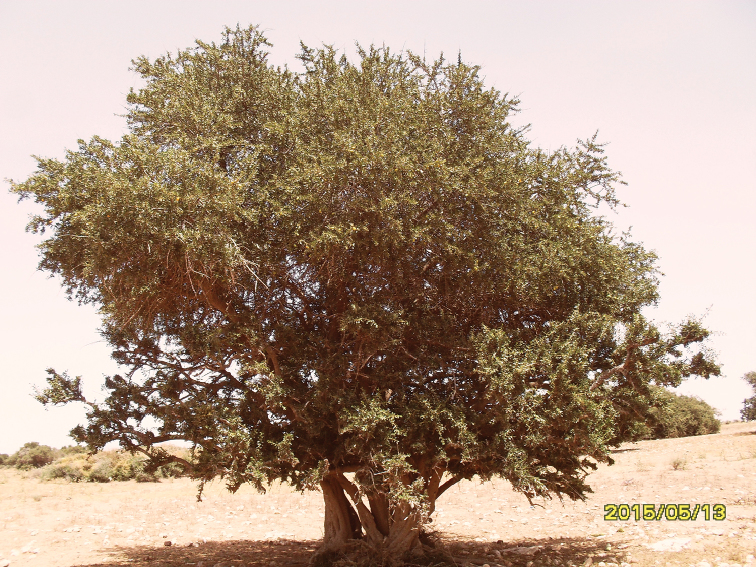
*Argania spinosa* L. host plant of *Ceratitis capitata*.

A highly polyphagous species, including the infestation of fruits of *Ficus carrica* L., *Prunus armeniaca* L., *Prunus persica* L., and *Pyrus communis* L. from which the recorded specimens were reared, and *Argania spinosa* L. (Fig. 1) from which our specimens were collected by sweep-netting.

######### World distribution.

Algeria, Tunisia, Libya, Egypt, Angola, Burundi, Cameroon, Congo, Ghana, Guinea, Kenya, Madagascar, Mozambique, Nigeria, South Africa, Seychelles Is, St Helena I., Tanzania, Benin, Burkino Faso, Cape Verde Is, Ethiopia, Gabon, Ivory Coast, Liberia, Malawi, Mali, Mauritius I., Niger, Reunion I., São Tomé I., Senegal, Sudan, Togo, Uganda, Zimbabwe, South Europe, Middle East, Neotropics, West Australia, Hawaii (Norrbom et al 1999, De Meyer 2000, De Meyer et al. 2013).

###### Tribe DACINI Loew, 1862

####### Genus *BACTROCERA* Macquart, 1835

######## Subgenus Bactrocera Macquart, 1835

######### Bactrocera oleae

Taxon classificationAnimaliaDipteraTephritidae

(Rossi, 1790)

########## Literature record.

Morocco, Rif: Tangier (Becker and Stein 1913). First record for the Middle and Anti Atlas.

########## New records.

Rif: El Haouta, 1♂, 30-I-2014 (net sweeping); Oued Maâza (Tarik El Ouasâa), 5♂♂, 1♀ 18-III-2015 (reared); Cascade Chrafate, 1♂, 28-IV-2015 (net sweeping); Koudia El Aouinate, 3♂♂, 6♀♀, 12-XII-2015 (net sweeping); Lâazaba, 2♂♂, 12-XII-2015 (net sweeping); Dhar Sbagh Mâasra, 2♂♂, 12-XII-2015 (net sweeping); El Hajria, 1♀, 13-XII-2015 (net sweeping). Middle Atlas: Sensla, 1♂, 1♀, 14-VII-2016 (net sweeping). Anti Atlas: Route Bab El Khemis, 2♂♂, 2♀♀, 14-V-2015 (net sweeping).

########## Host plant.

Fruits of *Olea europea* L. from which the specimens were both reared and collected by sweep-netting.

########## World distribution.

Mediterranean region, Eritrea, Ethiopia, Kenya, Angola, Lesotho, Namibia, South Africa, Sudan, Greece, Italy, Portugal, Spain, Madeira, Canary Islands, North Africa, Israel, Lebanon, Jordan, Réunion, Saudi Arabia, Syria, Turkey, Caucasus, Pakistan, nw. India, USA, Mexico (Séguy 1930, Munro 1984, White and Hancock 1997, Norrbom et al. 1999, Copeland et al. 2004, White 2006).

####### Genus *DACUS* Fabricius, 1805

######## Dacus frontalis

Taxon classificationAnimaliaDipteraTephritidae

(Becker, 1922)

######### New records.

Morocco, Anti Atlas: Oued Foum Ziguid (Douar Ouaiftoute), 1♂, 2♀♀, 01-VI-2015 (net sweeping); Oued Drâa (Ikhf Mezrou), 1♂, 1♀, 06-VI-2015 (net sweeping); Isdaoun, 2♂♂, 3♀♀, 07-VI-2015 (net sweeping). First record for Morocco.

######### World distribution.

Algeria, Libya, Egypt, Angola, Benin, Botswana, Cape Verde Is., Congo, Eritrea, Kenya, Lesotho, Namibia, Saudi Arabia, South Africa, Sudan, Tanzania, Yemen, Zimbabwe (White 2006, White and Goodger 2009, De Meyer et al. 2013).

######## Dacus longistylus

Taxon classificationAnimaliaDipteraTephritidae

(Wiedemann, 1830)

######### New records.

Morocco, Anti Atlas: Oued Tata, 1♀, 29-V-2015 (net sweeping); Douar Tighrimt, 1♂, 29-V-2015 (net sweeping); Oued Foum Ziguid (Douar Ouaiftoute), 31♂♂, 12♀♀, 01-VI-2015 (net sweeping). First record for Morocco.

######### Host plant.

**Figure 2.**
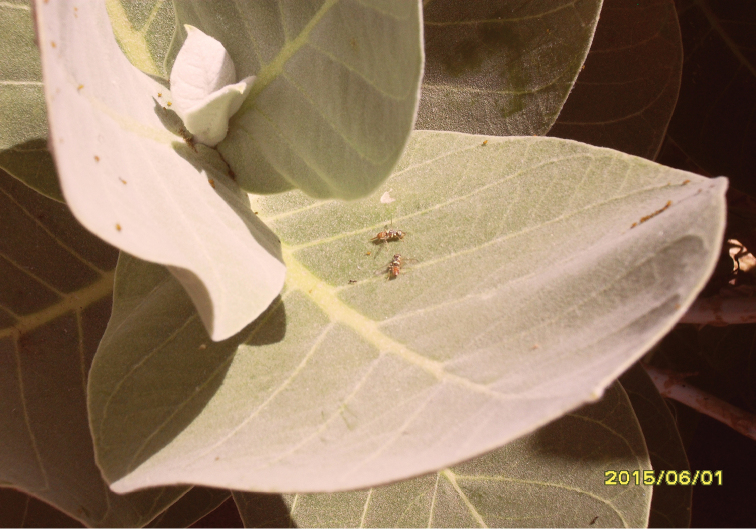
*Calotropis procera* L., host plant of *Dacus longistylus*.

*Calotropis* L. (Fig. 2) from which the specimens were collected by sweep-netting.

######### World distribution.

Libya, Egypt, Benin, Cameroon, Chad, Eritrea, Ethiopia, Kenya, Mali, Mauritania, Niger, Nigeria, Oman, Saudi Arabia, Senegal, Somalia, Sudan, Tanzania, Uganda, Yemen (White and Hancock 1997, White 2006, White and Goodger 2009, De Meyer et al. 2013).

#### Subfamily TEPHRITINAE (Newman, 1834)

##### Tribe DITHRYCINI Hendel, 1927

###### Subtribe OEDASPIDINA Hering, 1947

####### Genus *OEDASPIS* Loew, 1862

######## Oedaspis daphnea

Taxon classificationAnimaliaDipteraTephritidae

(Séguy, 1930)

######### Literature records.

Morocco, Middle Atlas: El Mers (Séguy 1930, Foote 1984, Norrbom et al. 1999).

######### World distribution.

######### Comments.

The species was described from Morocco and never recorded since; we did not examine the type material.

######## Oedaspis multifasciata

Taxon classificationAnimaliaDipteraTephritidae

(Loew, 1850)

######### Literature records.

Morocco, Middle Atlas: Itzer (Séguy 1953).

######### World distribution.

Spain, France, Germany, Italy, Croatia, Austria, Ukraine (Korneyev 2002).

######## Oedaspis trotteriana

Taxon classificationAnimaliaDipteraTephritidae

(Bezzi, 1913)

######### Literature records.

Morocco (Ribera and Blasco-Zumeta 1998).

######### World distribution.

Algeria, Libya, Egypt, Israel (Ribera and Blasco-Zumeta 1998, Norrbom et al. 1999).

##### Tribe MYOPITINI Hendel, 1927

###### Genus *MYOPITES* Blot, 1827

####### Myopites inulaedyssentericae

Taxon classificationAnimaliaDipteraTephritidae

Blot, 1827

######## New record.

Morocco, Rif: Affluent Tarmast, 1♂, 26-IV-2013; Aïn Afersiw, 1♀, 11-VI-2013 (net sweeping). First record for Morocco.

######## World distribution.

Algeria, Great Britain, Estonia, France, Germany, Italy, Poland, Russia, Spain, Switzerland, Ukraine, Balkans, central Europe (Evstigneev 2011).

####### Myopites stylatus

Taxon classificationAnimaliaDipteraTephritidae

Fabricius, 1794

######## New record.

Morocco, Rif: Affluent Tarmast, 1♀, 26-VI-2013; Oued El Hamma, 1♂, 25-IV-2014; El Haouta, 1♂, 18-VI-2014 (net sweeping). First record for Morocco.

######## World distribution.

North Africa, southern Europe, Israel, Spain, Albania, (Zimsen 1964, Freidberg and Kugler 1989, Norrbom et al 1999, Merz 2001, Korneyev 2003).

###### Genus *UROPHORA* Robineau-Desvoidy, 1830

####### Subgenus Urophora Robineau-Desvoidy, 1830

= *Euribia* Meigen, 1800

######## Urophora sp. near congrua

Taxon classificationAnimaliaDipteraTephritidae

######### Literature records.

Morocco, Anti Atlas: Taroudant (Séguy 1941).

######### World distribution.

France, South Germany, Austria (Norrbom et al, 1999).

######### Comments.

The presence of *Urophora congrua* Loew, 1862 in Morocco is obviously a misidentification of another *Urophora* (Personal communication with Valery Korneyev). The literature records and the world distribution given above are those of *U. congrua* and therefore do not necessarily represent the true information for the species that occurs in Morocco

######## Urophora mauritanica

Taxon classificationAnimaliaDipteraTephritidae

(Macquart, 1851)

######### Literature records.

Morocco, High Atlas: Imi N’Takandout, Dar Kaid M’tougui (Séguy 1930), Ito (White and Korneyev 1989). First record for the Anti Atlas.

######### New records.

Morocco, Anti Atlas: Airport Sidi Ifni, 1♀, 17-V-2015; Oued Tata, 5♂♂, 29-V-2015 (net sweeping); Oued Tata, 12♂♂, 8♀♀, 06-VI-2015 (reared); Douar Tighrimt, 3♂♂, 29-V-2015; Imzine, 3♂♂, 1♀, 30-V-2015; Jnane Makadir, 1♀, 03-VI-2015; Oued Ouarzazate, 2♂♂, 12-VI-2015 (net sweeping)- 25♂♂, 14♀♀, 28-VI-2015- 7♂♂, 2♀♀, 01-VII-2015 (reared).

######### Host plant.

**Figure 3.**
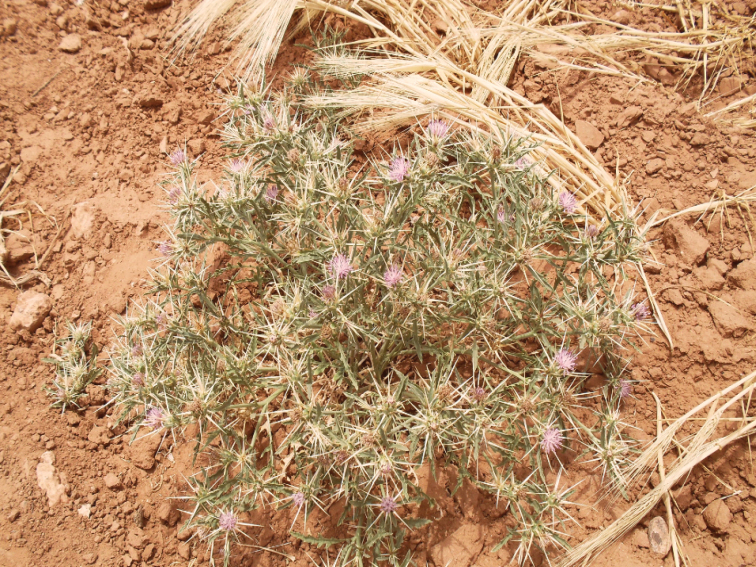
*Centaurea calcitrapa* L., host plant of *Urophora mauritanica*, *Chaetorellia conjuncta* and *Terellia virens*.

*Centaurea calcitrapa* L. (Fig. 3) from which the specimens were reared.

######### World distribution.

Algeria, Libya, Albania, Azerbaijan, Bulgaria, Cyprus, Czech Rep., France, Greece, Iran, Israel, Italy, Jordan, Kazakhstan, Lebanon, Macedonia, Malta, Moldova, Russia, Spain, Tajikistan, Turkey, Turkmenistan, Ukraine, Uzbekistan (White and Korneyev 1989, Korneyev and White 1996, 1999, Norrbom et al. 1999, Gharali et al. 2005, Khaghaninia and Gharajedaghi 2012).

######## Urophora quadrifasciata

Taxon classificationAnimaliaDipteraTephritidae

(Meigen, 1826)

######### Literature records.

Morocco (Séguy 1930).

######### World distribution.

South to North Africa, Europe, Kazakstan, Iran, North America, Australia (Norrbom et al. 1999, Heřman and Dirlbek 2006).

######## Urophora solstitialis

Taxon classificationAnimaliaDipteraTephritidae

Linnaeus, 1758

######### Literature record.

Morocco, High Atlas: Haute Reraya (Séguy 1930).

######### World distribution.

Tunisia, Great Britain, Eastern Scandianvia to Kazakhstan, China, France, Italy, Switzerland, Turkey, Iran, Armenia, Moldova, Poland, Russia, Ukraine, North America, Australia, New Zealand (Korneyev and White 1999, Heřman and Dirlbek 2006, Evstigneev 2011).

##### Tribe Noeetini (Norrbom & Korneyev, 1999)

###### Genus *ENSINA* Robineau-Desvoidy, 1830

####### Ensina sonchi

Taxon classificationAnimaliaDipteraTephritidae

(Linnaeus, 1767)

######## New records.

Morocco, Rif: Ksar Rimal, 5♂♂, 2♀♀, 05-VI-2013- Douar Tizga, 1♂, 25-VI-2014- Oued El Kebir, 1♀, 31-V-2015. Middle Atlas: Sensla, 1♂, 14-VII-2016 (net sweeping). Anti Atlas: Oued Massa (Pont Aghbalou), 1♀, 12-V-2015 (net sweeping); Centre Sidi Ouassay, 1♂, 12-V-2015; Aïn Boharroch, 1♀, 13-XII-2015; Atbane, 1♀, 14-V-2015; Oued Tamanarne, 1♂, 26-V-2015; Oued Drâa (Tahtah), 6♂♂, 7♀♀, 03-VI-2015; Jnane Makadir, 3♂♂, 1♀, 03-VI-2015; Kasbah Asma, 16♂♂, 13♀♀, 04-VI-2015 (net sweeping)- 7♂♂, 13♀♀, 10-VI-2015 (reared); Aït Aissa O Brahim, 1♀, 05-VI-2015; Oued Ziz (Pont Errachidia), 2♂♂, 2♀♀, 09-VI-2015; Oued Ouarzazate, 4♂♂, 2♀♀, 12-VI-2015 (net sweeping). First record for Morocco.

######## Host plants.

**Figure 4.**
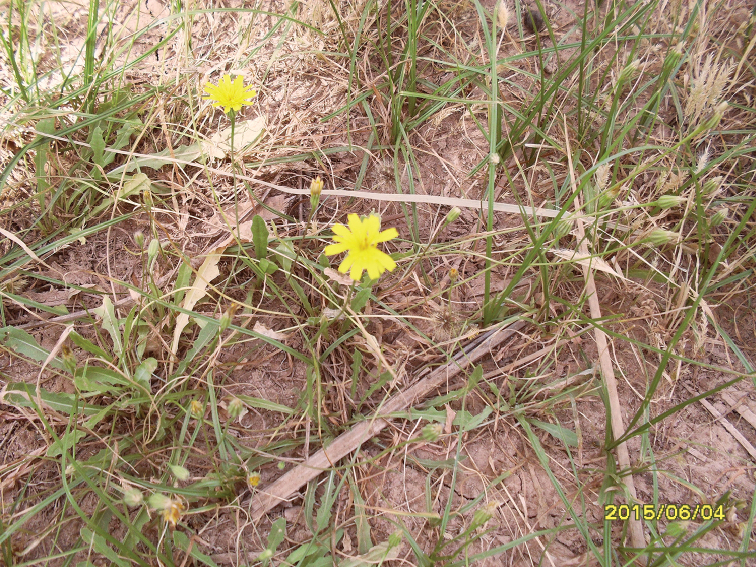
*Hypochaeris* sp., host plant of *Ensina sonchi*.

Flower heads of *Hypochaeris radicata* L. (Fig. 4) from which the specimens were reared.

######## World distribution.

South to North Africa, Great Britain, Scandinavia, Andorra, Czech Rep., France, Germany, Iran, Poland, Portugal, Spain, Sweden, Switzerland, Turkey, Ukraine, Saudi Arabia, Japan, Korea, India, Ethiopia, Taiwan, Philippines, Hawaii (White and Elson-Harris 1992, Merz and Dawah 2005, Heřman and Dirlbek 2006, Gharajedaghi et al. 2012).

###### Genus *HYPENIDIUM* Loew, 1862

####### Hypenidium graecum

Taxon classificationAnimaliaDipteraTephritidae

(Loew, 1862)

= Stephanaciura bipartita Séguy, 1930

######## Literature records.

Morocco, Middle Atlas: Taffert (Séguy 1930, Norrbom et al. 1999).

######## World distribution.

Bosnia, Greece, Hungary, Israel, Italy, Portugal, Spain, Ukraine (Korneyev et al. 2011).

##### Tribe TEPHRELLINI Hendel, 1927

###### Subtribe TEPHRELLINA Hendel, 1927

####### Genus *ACIURA* Robineau-Desvoidy, 1830

######## Aciura coryli

Taxon classificationAnimaliaDipteraTephritidae

(Rossi, 1794)

= Aciura powelli (Séguy, 1930)

######### Literature records.

Morocco, Middle Atlas: Azrou (Séguy 1930), Korifla (Séguy 1953).

######### World distribution.

North Africa, Spain and southern France to southern Ukraine, Canary Islands, Esrael, Syria (Korneyev and Dirlbek 2000).

####### Genus *OXYACIURA* Hendel, 1927

######## Oxyaciura tibialis

Taxon classificationAnimaliaDipteraTephritidae

(Robineau-Desvoidy, 1830)

######### Literature records.

Morocco, Rif: Tangier (Becker and Stein 1913), Sker (Séguy 1930).

######### New sites.

Morocco, Rif: Daya El Birdiyel, 1♂, 27-VI-2013; Oued Azila, 1♂, 27-VI-2013; Maison forestière, 1♂, 17-VI-2014; Oued Maâza (Tarik El Ouasâa), 1♂, 18-IV-2015 (net sweeping).

######### World distribution.

North Africa, Canary Islands, Cape Verde Islands, Madeira, Spain, China, Iran, Israel, Kazakhstan, Saudi Arabia, Syria, United Arab Emirates, Afghanistan, Ethiopia (Merz 1992, 2008, Korneyev and Dirlbek 2000, Merz and Dawah 2005, Gharajedaghi et al. 2012).

####### Genus *SPHAENISCUS* Becker, 1908

######## Sphaeniscus filiolus

Taxon classificationAnimaliaDipteraTephritidae

(Loew, 1869)

= Spheniscomyia aegyptiaca (Efflatoun, 1924)

######### Literature records.

Morocco, Anti Atlas: Goulimine (Séguy 1930, 1949). First record for the Rif.

######### New records.

Morocco, Rif: Affluent Tarmast, 1♂, 26-VI-2013; Oued Maâza (Tarik El Ouasâa), 2♂♂, 1♀, 19-VI-2014 (net sweeping).

######### World distribution.

Egypt, Spain, Canary Islands, Cape Verde Islands, Israel, Ethiopia (Séguy 1930, 1949; Merz 1992, 2001).

##### Tribe TEPHRITINI Macquart, 1835

###### Genus *ACANTHIOPHILUS* Becker, 1908

####### Acanthiophilus helianthi

Taxon classificationAnimaliaDipteraTephritidae

(Rossi, 1794)

######## Literature records.

Morocco, Rif: around Tangier (Becker and Stein 1913, Séguy 1930), Middle Atlas (Séguy 1930), Anti Atlas: Goulimine, Foum el Hassan, Alnif, Tarfaia, Boufarik (Séguy 1949).

######## New sites.

Morocco, Rif: Oued Zinat, 1♀, 27-IV-2012; Ksar Rimal, 1♀, 10-V-2013; Oued Derdara, 1♀, 24-V-2013; Affluent Tarmast, 1♂, 26-VI-2013; Daya El Birdiyel, 1♀, 27-VI-2013; Oued Azila, 1♂, 27-VI-2013; Daya Jbel Zemzem, 1♂, 23-IV-2014; Oued Boumarouil, 2♂♂, 1♀, 10-V-2014; Douar Abou Boubnar (Marabout Sidi Gile), 1♀, 18-V-2014; El Haouta, 1♂, 18-VI-2014; Oued Maâza (Tarik El Ouasâa), 1♀, 19-VI-2014- 1♀, 18-IV-2015; Douar Tizga, 1♂, 25-VI-2014; Daya Afrate, 1♀, 18-IV-2015; Oued Mezine, 1♂, 18-IV-2015; Aïn El Malâab, 1♂, 1♀, 21-IV-2015; Oued Jnane Niche, 1♀, 25-IV-2015. Middle Atlas: Oued Oum Er Rbia, 1♂, 14-VII-2016. Anti Atlas: Centre Sidi Ouassay, 1♂, 2♀♀, 12-V-2015; Avant Sidi Binzarne, 1♀, 12-V-2015; Route Bab El Khemis, 1♂, 1♀, 14-V-2015; Airport Sidi Ifni, 22♂♂, 22♀♀, 17-V-2015; Oued Tisla, 1♀, 24-V-2015; Oued Sayad, 1♀, 25-V-2015; Oued Tamanarne, 1♂, 1♀, 26-V-2015; Oued Foum Ziguid (Douar Ouaiftoute), 1♂, 1♀, 01-VI-2015; Jnane Makadir, 2♂♂, 4♀♀, 03-VI-2015; Douar Rggaga, 1♀, 05-VI-2015; Oued Tinghir, 1♂, 1♀, 11-VI-2015; Oued Ouarzazate, 12♂♂, 6♀♀, 12-VI-2015 (net sweeping).

######## World distribution.

Algeria, Tunisia, Albania, Andorra, Austria, Balearic Is., Belgium, Great Britain, Bulgaria, Corsica, Cyprus, Czech Rep., France, Germany, Greece, Hungary, Italy, Malta, Moldova, Netherlands, Norway, Poland, Portugal, Romania, Russia, Sardinia, Sicily, Slovakia, Spain, Switzerland, Ukraine, Turkey, Central Asia, Mongolia, China, Madeira, Canary Islands, Ethiopia, Kenya, Sudan, Afghanistan, Iran, Israel, Kazakhstan, Kyrgyzstan, Lebanon, Saudi Arabia, United Arab Emirates, Syria, India, Nepal, Pakistan, Thailand (Séguy 1930, Merz and Korneyev 2004, Heřman and Dirlbek 2006, David and Ramani 2011, Merz 2011).

###### Genus *CAMPIGLOSSA* Rondani, 1870

####### Campiglossa martii

Taxon classificationAnimaliaDipteraTephritidae

(Becker, 1908)

= Oxyna martii Becker, 1908

######## New records.

North Africa, Morocco, Rif: Oued El Kebir, 1♀, 31-V-2014 (net sweeping). Anti Atlas: Centre Sidi Ouassay, 1♂, 12-V-2015 (net sweeping). First record for Morocco and North Africa.

######## World distribution.

Canary Islands (Frey 1958a), Cape Verde Islands (Frey 1958b), Spain (Merz 1992).

####### Campiglossa producta

Taxon classificationAnimaliaDipteraTephritidae

Loew, 1844

= Paroxyna tessellata Hendel, 1927

######## Literature records.

Morocco, Rif: Tangier and its surroundings, (Becker and Stein 1913, Séguy 1930), High Atlas (Séguy 1930).

######## New sites.

Morocco, Rif: Oued Al Mizzine, 1♀, 11-VI-2013; Aïn El Malâab, 1♂, 17-V-2014; Daya El Hajjami, 1♂, 28-III-2015 (net sweeping).

######## World distribution.

Algeria, Tunisia, Great Britain, Finland, Netherlands, Poland, Portugal, Spain, Switzerland, Madeira, Canary Islands, Turkey, Syria, Jordan, Iran, Israel, Iraq, Afghanistan, Central Asia, India, China, Thailand, Vietnam, Korea, Japan (Korneyev and Dirlbek 2000, Hancock and McGuire 2002, Korneyev 2004, Heřman and Dirlbek 2006, Mohamadzade Namin and Nozari 2011).

###### Genus *CAPITITES* Foote & Freidberg, 1981

####### Capitites ramulosa

Taxon classificationAnimaliaDipteraTephritidae

(Loew, 1844)

######## Literature records.

Morocco, Middle Atlas: Forest of Timelilt (Séguy 1941). High Atlas: Tizi n’Test, Imdress, Taroudant (Séguy 1941), Foum El Hassan, Akka, Agdz, Alnif (Séguy 1949).

######## World distribution.

Algeria, Tunisia, Egypt, Greece, Portugal, Spain, Cyprus, Syria, Israel, Iraq, Canary Islands, Cape Verde Islands (Séguy 1930, Freidberg and Kugler 1989, Merz 1992, Norrbom et al. 1999, Korneyev and Dirlbek 2000).

###### Genus *DESMELLA* Munro, 1957

####### Desmella rostellata

Taxon classificationAnimaliaDipteraTephritidae

(Séguy, 1941)

= Paroxyna rostellata (Séguy, 1941)

######## Literature record.

Morocco, Anti Atlas: Agadir (Séguy 1941).

###### Genus *DIOXYNA* Frey, 1945

####### Dioxyna sororcula

Taxon classificationAnimaliaDipteraTephritidae

(Wiedemann, 1830)

######## New records.

Morocco, Rif: Ksar Rimal, 2♂♂, 19-V-2013- 1♂, 2♀♀, 26-V-2013; Oued Jnane Niche, 2♀♀, 14-VI-2013; Oued Halila, 1♂, 05-XI-2013; Oued Zarka, 7♂♂, 1♀, 14-XI-2013; Oued Martil, 1♂, 1♀, 13-XII-2013; Oued Amsa, 1♀, 19-XII-2013; Oued Sahel, 1♂, 05-IV-2014; Daya Jbel Zemzem, 1♂, 17-IV-2014; Oued Majjou, 2♂♂, 10-V-2014; Dhar Sbagh Mâasra, 1♀, 12-XII-2015; Douar Kitane, 15♂♂, 5♀♀, 14-XI-2013- 1♂, 2♀♀, 13-III-2014- 1♀, 17-III-2014- 2♂♂, 1♀, 10-IV-2014- 5♂♂, 13♀♀, 02-V-2014- 26♂♂, 14♀♀, 23-I-2016 (net sweeping). First record for Morocco.

######## World distribution.

Algeria, Tunisia, South Africa, Madeira, Canary Islands, Spain, Saudi Arabia, Yemen, India, West Bengal, Nepal, Thailand, China, Korea, Japan, Taiwan, Botswana, Namibia, Zambia, Zimbabwe, Australia, New Caledonia, Western Samoa, Niue, Cook Is., French Polynesia, Fiji, Hawaii (Hancock and McGuire 2001, Norrbom and Hancock 2004, Merz and Dawah 2005, David and Ramani 2011).

###### Genus *EUARESTA* Loew, 1873

####### Euaresta bullans

Taxon classificationAnimaliaDipteraTephritidae

(Wiedemann, 1830)

######## Literature records.

Morocco, Anti Atlas: Tiznit, Sidi-Moussa-d’Aglou (Heřman and Dirlbek 2006).

######## New site.

Morocco, Anti Atlas: Msidira, 2♂♂, 1♀, 18-V-2015 (net sweeping).

######## World distribution.

Algeria, Tunisia, South Africa, Argentina, Bolivia, Chile, Peru, Uruguay, California, Arizona, Bulgaria, France, Greece, Hungary, Iran, Israel, Italy, Macedonia, Moldova, Russia, Slovakia, Spain, Turkey, Ukraine, Australia (Herman and Dirlbek 2006, Khaghaninia and Gharajedaghi 2012).

###### Genus *GONIURELLIA* Hendel, 1927

####### Goniurellia longicauda

Taxon classificationAnimaliaDipteraTephritidae

(Freidberg, 1980)

######## Literature records.

Morocco, Middle Atlas: Tizi s’Tkrine, Forest of Azrou (Séguy 1930, Freidberg 1980), High Atlas: Taroudant (Séguy 1953, Freidberg 1980). Anti Atlas: Edehby Ouarzazate (Pârvu et al. 2006).

######## New sites.

Morocco, Anti Atlas: Airport Sidi Ifni, 4♂♂, 17-V-2015; Oued Tisla, 6♂♂, 9♀♀, 24-V-2015; Oued Tamanarne, 1♂, 26-V-2015; Douar Zaouiet, 1♂, 27-V-2015; Oued Tata, 1♀, 29-V-2015; Douar Tighrimt, 1♀, 29-V-2015; Ksibat Elhdeb, 2♂♂, 07-VI-2015; Oued Ziz (Pont Errachidia), 1♀, 09-VI-2015; Oued Ouarzazate, 2♂♂, 12-VI-2015 (net sweeping).

######## World distribution.

Algeria, Tunisia, Libya, Egypt, France, Canary Islands, Cape Verde Islands, Iran, Iraq, Israel, Saudi Arabia, Syria, Turkey, United Arab Emirates, Kenya (Freidberg 1980, Freidberg and Kugler 1989, Merz 1992, Norrbom et al. 1999, 2008, Korneyev and Dirlbek 2000, Al Dhafer et al. 2012).

####### Goniurellia persignata

Taxon classificationAnimaliaDipteraTephritidae

Freidberg, 1980

######## Literature records.

Morocco, Eastern region: Defilia, nr. Figuig (Freidberg 1980). Anti Atlas: Tiffoultoute (Norrbom et al. 1999, Heřman and Dirlbek 2006).

######## New records.

Morocco, Rif: Dhar Sbagh Mâasra, 1♀, 12-XII-2015. Anti Atlas: Douar Zaouiet, 1♂, 27-V-2015; Oued Ouarzazate, 1♂, 12-VI-2015 (net sweeping). First record for the Rif.

######## World distribution.

Egypt, China, Crete, Cyprus, Ethiopia, Israel, Saudi Arabia, Sri Lanka, Turkmenistan, Namibia? (Freidberg 1980, Norrbom et al. 1999, Hancock et al. 2001, Merz and Dawah 2005, Herman and Dirlbek 2006).

###### Genus *SPATHULINA* Rondani, 1856

####### Spathulina sicula

Taxon classificationAnimaliaDipteraTephritidae

(Rondani, 1856)

######## Literature records.

Morocco (Séguy 1930).

######## New site.

Morocco, Rif: Barrage Smir, 1♂, 27-IV-2014 (net sweeping).

######## World distribution.

Spain, Portugal, Italy, Israel, Canary Is. (Norrbom et al. 1999).

###### Genus *SPHENELLA* Robineau-Desvoidy, 1830

####### Sphenella marginata

Taxon classificationAnimaliaDipteraTephritidae

(Fallén, 1814)

######## Literature records.

Morocco, Rif: around Tangier (Becker and Stein 1913, Séguy 1930).

######## New sites.

Morocco, Rif: Affluent Tarmast, 1♂, 26-VI-2013; Daya Jbel Zemzem, 5♂♂, 2♀♀, 23-IV-2014; El Malâab, 1♂, 02-II-2015; Oued Maâza (Âachira), 1♀, 10-III-2015; Daya Aïn Jdioui, 1♀, 28-III-2015; Oued Majjou, 1♀, 09-IV-2015; Daya Afrate, 1♂, 1♀, 18-IV-2015 (net sweeping).

######## World distribution.

Egypt, South Africa, Czech Rep., Poland, Portugal, Spain, Sweden, Switzerland, Ukraine, Russia, Madeira, Afghanistan, Canary Islands, China, Iran, Israel, Kazakhstan, Saudi Arabia, Turkey, Turkmenistan, Eritrea, Kenya, Lesotho, Mozambique (Gharajedaghi et al. 2012).

###### Genus *TEPHRITIS* (Latreille, 1804)

####### Tephritis dioscurea

Taxon classificationAnimaliaDipteraTephritidae

Loew, 1856

######## Literature records.

Morocco, Middle Atlas: El Hajeb (Séguy 1930).

######## World distribution.

Sweden, France, Hungary, Austria, Germany, Switzerland, Russia, Estonia, Latvia, Lithuania, Ukraine, Moldova, Azerbaijan, Armenia, Georgia, Kazakhstan, Turkey, Iran (Foote 1984, Merz 1994, Thompson 1998, Kütük 2005, Zarghani et al. 2010).

####### Tephritis divisa

Taxon classificationAnimaliaDipteraTephritidae

(Rondani, 1871)

######## New record.

Morocco, Rif: Daya Amsemlil, 1♀, 01-XI-2014 (net sweeping). First record for Morocco and North Africa.

######## World distribution.

Croatia, France, Greece, Israel, Italy, Near East Asia, Spain, Switzerland, Turkey and Ukraine (Norrbom 1999, Korneyev 2003, Merz and Korneyev 2004, Kütük 2005).

####### Tephritis formosa

Taxon classificationAnimaliaDipteraTephritidae

(Loew, 1844)

######## Literature record.

Morocco, High Atlas: Asni (Séguy 1930).

######## New records.

Morocco, Rif: Oued Abou Bnar, 1♂, 18-V-2014; Oued Sidi Ben Sâada, 1♂, 30-V-2014; Oued Achekrade, 1♂, 31-V-2014; Oued El Kanar, 1♀, 25-IV-2015 (net sweeping). First record for the Rif.

######## World distribution.

Caucasus, Israel, Persia, Russia, Ukraine, Moldova, Azerbaijan, Georgia, Armenia, England, Switzerland, Germany, Iran, Turkey, China (Hendel 1927, Dirlbek and Dirlbek 1971, Giray 1979, Foote 1984, White 1988, Freidberg and Kugler 1989, Merz 1994, Wang 1998).

####### Tephritis leontodontis

Taxon classificationAnimaliaDipteraTephritidae

De Géer, 1776

######## Literature record.

Morocco (Séguy 1930).

######## World distribution.

Austria, Albania, Belgium, Bulgaria, United Kingdom, Estonia, Ireland, Spain, Italy, Latvia, Lithuania, Netherlands, Germany, Norway, Poland, Russian Federation, Romania, Slovakia, Hungary, Ukraine, Finland, France, United Kingdom, Czech Republic, Sweden, Switzerland (Korneyev 2016).

####### Tephritis matricariae

Taxon classificationAnimaliaDipteraTephritidae

Loew, 1844

######## Literature records.

Morocco (Séguy 1930).

######## New sites.

Morocco, Rif: Affluent Oued Amsemlil, 1♂, 30-V-2013; El Haouta, 1♀, 30-I-2014; Daya Jbel Zemzem, 1♂, 23-IV-2014; Daya Amsemlil, 1♂, 01-XI-2014; Oued El Hamma, 1♀, 24-IV-2015 (net sweeping).

######## World distribution.

Algeria, Tunisia, Libya, Egypt, England, Netherlands, Austria, Andorra, Greece, Portugal, Spain, Switzerland, Turkey, Iran (Norrbom et al. 1999, Merz 2001, Mohamadzade Namin et al. 2010a).

####### Tephritis nigricauda

Taxon classificationAnimaliaDipteraTephritidae

Loew, 1856

######## Literature records.

Morocco: Berrechid (Séguy 1930). First record for the Rif.

######## New sites.

Daya Jbel Zemzem, 2♂♂, 23-IV-2014; Oued Maâza (Tarik El Ouasâa), 1♂, 18-IV-2015; Aïn El Maounzil, 1♂, 21-IV-2015; Daya Tazia, 1♀, 24-IV-2015; Daya Amsemlil, 1♀, 27-V-2015; Douar Tamakoute, 1♂, 1♀, 25-XII-2015 (net sweeping).

######## World distribution.

Algeria, Egypt, Poland, Portugal, Spain, Iran, Iraq, Jordan, Russia, Syria (Korneyev and Dirlbek 2000, Merz 2001, Gharajedaghi et al. 2012).

####### Tephritis postica

Taxon classificationAnimaliaDipteraTephritidae

(Loew, 1844)

######## Literature records.

Morocco, Middle Atlas: Volubilis (Heřman and Dirlbek 2006). First record for the Anti Atlas.

######## New records.

Anti Atlas: Ksibat Elhdeb, 1♀, 07-VI-2015 (net sweeping)- 1♂, 1♀, 23-VI-2015- 1♂, 1♀, 26-VI-2015- 1♂, 1♀, 28-VI-2015 (reared); Oued Tinghir, 1♂, 11-VI-2015 (net sweeping).

######## Host plant.

**Figure 5.**
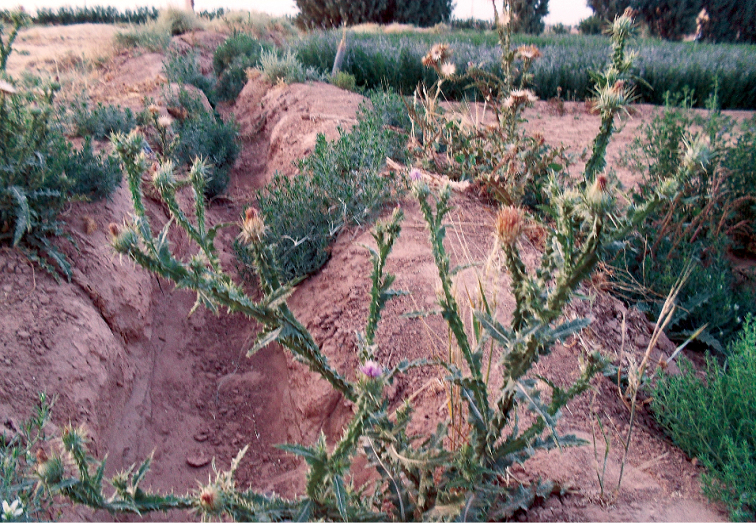
*Onopordum acanthium* L. host plant of *Tephritis postica*.

Flower heads of *Onopordum acanthium* L. (Fig. 5) from which the specimens were reared.

######## World distribution.

Algeria, Tunisia, Spain, France, Czech Rep., Georgia, Germany, Hungary, Poland, Slovakia, Ukraine, Russia, Turkey, Kazakhstan, Kyrgyzstan, China, Iran, Israel, Lebanon (Séguy 1934a, Richter 1970, Merz 1994, 2001, Wang 1998, Klasa 2001, Heřman and Dirlbek 2006, Zarghani et al. 2010, Gharajedaghi et al. 2012, Hancock 2013).

####### Tephritis praecox

Taxon classificationAnimaliaDipteraTephritidae

(Loew, 1844)

######## Literature records.

Morocco, Rif: Tangier (Heřman and Dirlbek 2006). Middle Atlas: Tizi s’Tkrine (Séguy 1930), Ifrane-Azrou National Park (Heřman and Dirlbek 2006). First record for eastern Morocco.

######## New sites.

Morocco, Rif: Daya El Ânassar, 2♂♂, 24-V-2013; Daya Amsemlil, 1♀, 30-V-2013; Affluent Oued Amsemlil, 1♀, 30-V-2013; Douar Dacheryène, 1♀, 04-IV-2014; Douar Taghbaloute, 1♂, 05-IV-2014; Barrage Nakhla, 1♂, 05-IV-2014; Oued Sahel, 2♂♂, 05-IV-2014; Daya Jbel Zemzem, 4♂♂, 2♀♀, 17-IV-2014- 1♂, 23-IV-2014; Douar Kitane, 1♂, 19-IV-2014; Oued El Hamma, 1♂, 25-IV-2014; Oued El Kebir, 2♂♂, 2♀♀, 25-IV-2014; Aïn El Ma Bared, 1♂, 06-V-2014; Aïn El Malâab, 1♂, 1♀, 17-V-2014; Douar Abou Boubnar (Marabout Sidi Gile), 1♀, 18-V-2014; Maison forestière, 1♂, 1♀, 17-VI-2014; Douar Tizga, 1♀, 25-VI-2014; Oued Aïn Jdioui (Touaret), 1♀, 28-III-2015; Daya Afrate, 1♂, 18-IV-2015; Oued Jbara, 1♂, 18-IV-2015; Aïn El Malâab, 1♂, 21-IV-2015; Aïn El Maounzil, 11♂♂, 8♀♀, 21-IV-2015; Daya Tazia, 1♂, 24-IV-2015; Oued Jnane Niche, 5♂♂, 4♀♀, 25-IV-2015; Oued Majjou, 1♀, 27-IV-2015; Aïn Tiouila, 1♂, 02-V-2015; Daya Mtahen, 1♂, 1♀, 07-V-2015; Lâazaba, 3♂♂, 1♀, 12-XII-2015; Dhar Sbagh Mâasra, 1♂, 3♀♀, 12-XII-2015; El Hajria, 1♀, 13-XII-2015; Aïn Boharroch, 1♂, 13-XII-2015; Douar Tamakout, 1♀, 25-XII-2015; Douar Ouslaf, 1♂, 2♀♀, 26-XII-2015 (net sweeping). Beni Snassen (Eastern Morocco): Oued Beni Ouaklane, 1♂, 24-XI-2014.

######## World distribution.

Algeria, Spain, Portugal, Switzerland, Great Britain, Austria, Hungary, Madeira, Canary Islands, Syria, Iran, Israel, Iraq, Afghanistan (Séguy 1930, Norrbom et al. 1999, Korneyev and Dirlbek 2000, Smit 2006, Heřman and Dirlbek 2006, Zarghani et al. 2010, Gharajedaghi et al. 2012).

####### Tephritis pulchra

Taxon classificationAnimaliaDipteraTephritidae

Loew, 1844

######## Literature records.

Morocco (Séguy 1930).

######## World distribution.

North Africa, France, Spain, Austria, Hungary, Italy, Greece, Poland, Iran, Turkey, China (Norrbom et al. 1999, Merz 2001, Freidberg and Kütük 2002, Mohamadzade Namin and Nozari 2011).

####### Tephritis simplex

Taxon classificationAnimaliaDipteraTephritidae

Loew, 1844

######## Literature records.

Morocco (Séguy 1930).

######## World distribution.

Algeria, Tunisia, Spain, Portugal, Crete, Turkey, Israel (Norrbom et al. 1999, Merz 2001).

####### Tephritis stictica

Taxon classificationAnimaliaDipteraTephritidae

Loew, 1862

######## Literature records.

Morocco: Rabat (Séguy 1930).

######## World distribution.

Algeria (Séguy 1930), southern Europe (Norrbom et al. 1999).

####### Tephritis theryi

Taxon classificationAnimaliaDipteraTephritidae

(Séguy, 1930)

######## Literature records.

Morocco, High Atlas: Asni (Séguy 1930, Norrbom et al. 1999).

####### Tephritis vespertina

Taxon classificationAnimaliaDipteraTephritidae

(Loew, 1844)

######## New record.

Morocco, Rif: Daya Mtahen, 1♀, 27-V-2015; Dhar Sbagh Mâasra, 1♂, 1♀, 12-XII-2015 (net sweeping). First record for Morocco.

######## World distribution.

South to North Africa, Andorra, Germany, Hungary, Poland, Portugal, Spain, Switzerland, Ukraine, Turkey (Merz 2001, Hendel 1927, Foote 1984, Norrbom et al. 1999, Merz 1994, Kütük and Özgür 2003).

###### Genus *TEPHRITOMYIA* Hendel, 1927

####### Tephritomyia lauta

Taxon classificationAnimaliaDipteraTephritidae

(Loew, 1869)

######## Literature records.

Morocco, High Atlas: Tachdirt (Séguy 1930); Tizi-n-Tichka (Morgulis 2015). First record for the Rif.

######## New records.

Morocco, Rif: Daya El Birdiyel, 1♂, 27-VI-2013; Daya Amsemlil, 1♀, 01-XI-2014; Lâazaba, 1♂, 12-XII-2105. Anti Atlas: Msidira, 1♂, 18-V-2015; Oued Ouarzazate, 1♂, 6♀♀, 12-VI-2015 (net sweeping).

######## World distribution.

Tunisia, Egypt, Cyprus, Greece, Iran, Iraq, Israel, Lebanon, Syria, Turkey (Freidberg and Kugler 1989, Norrbom et al. 1999, Korneyev and Dirlbek 2000, Mohamadzade Namin and Nozari 2011, Morgulis 2015).

###### Genus *TRUPANEA* (Schrank, 1795)

####### Trupanea amoena

Taxon classificationAnimaliaDipteraTephritidae

(Frauenfeld, 1857)

######## Literature records.

Morocco, Middle Atlas: Aïn Leuh (Séguy 1930). First record for the Rif.

######## New records.

Morocco, Rif: Ksar Rimal, 1♂, 05-VI-2013; Oued Jnane niche, 1♂, 14-VI-2013; Affluent Tarmast, 2♀♀, 26-VI-2013; Oued Martil (Tamouda), 1♀, 13-VII-2013; Oued Amsa, 1♂, 13-XII-2013; Oued El Hamma, 2♂♂, 1♀, 25-IV-2014; Oued Boumarouil, 1♀, 10-V-2014; Oued Sidi Yahya Aârab, 1♂, 25-IV-2015; Aïn Tiouila, 1♂, 02-V-2015. Anti Atlas: Oued Massa (Pont Aghbalou), 1♂, 12-V-2015; Centre Sidi Ouassay, 1♂, 12-V-2015; Avant Sidi Binzarne, 2♂♂, 1♀, 12-V-2015; Oued Tisla, 2♀♀, 24-V-2015; Douar Tighrimt, 1♂, 29-V-2015; Oued Draa (Tahtah), 1♂, 03-VI-2015; Jnane Makadir, 1♂, 03-VI-2015; Douar Rggaga, 1♀, 05-VI-2015; Aït Aissa O Brahim, 1♂, 05-VI-2015; Oued Drâa (Ikhf Mezrou), 1♂, 06-VI-2015; Isdaoun, 1♂, 07-VI-2015; Ksibat Elhdeb, 1♀, 07-VI-2015; Oued Tinghir, 2♂♂, 11-VI-2015 (net sweeping).

######## World distribution.

North Africa, Canary Islands, Ceylon, Ethiopia, England, Germany, India, Iran, Israel, Middle Asia, Middle and North Europe, Netherlands, Philippines, Saudi Arabia, Switzerland, Taiwan, Turkey, United Arab Emirates (Hendel 1927, Séguy 1930, Giray 1969, Foote 1984, White 1988, Freidberg and Kugler 1989, Merz 1994, Merz and Korneyev 2004, Merz and Dawah 2005, Merz 2008, Mohamadzade Namin et al. 2010b).

####### Trupanea guimari

Taxon classificationAnimaliaDipteraTephritidae

(Becker, 1908)

######## Literature records.

Morocco, Middle Atlas: Tizi s’Tkrine, Forest of Azrou (Séguy 1930). High Atlas: Tenfecht (Séguy 1930). First record for the Anti Atlas.

######## New records.

Morocco, Anti Atlas: Centre Sidi Ouassay, 4♂♂, 12-V-2015; Msidira, 1♂, 18-V-2015; Jnane Makadir, 2♂♂, 1♀, 03-VI-2015; Aït Aissa O Brahim, 1♂, 1♀, 05-VI-2015; Ksibat Elhdeb, 1♀, 07-VI-2015 (net sweeping).

######## World distribution.

Algeria, Spain, Canary Islands, Cape Verde Islands (Merz 1992, 1999).

####### Trupanea stellata

Taxon classificationAnimaliaDipteraTephritidae

(Fuessly, 1775)

######## Literature records.

Morocco, Middle Atlas: Forest of Timelilt (Séguy 1930), Anti Atlas: Goulimine (Séguy 1949). First record for the Rif.

######## New records.

Morocco, Rif: Mizoghar, 1♀, 06-V-2014; Oued Maâza (Tarik El Ouasâa), 1♂, 19-VI-2014; Daya Afrate, 1♀, 18-IV-2015; Aïn El Malâab, 1♀, 21-IV-2015; Oued Tkaraâ, 1♀, 07-V-2015. Anti Atlas: Centre Sidi Ouassay, 1♀, 12-V-2015 (net sweeping).

######## World distribution.

Tunisia, Andorra, Armenia, British Isles, Scandinavia, Czech Rep., Madeira, Poland, Spain, Switzerland, Turkey, Ukraine, Mongolia, China, Canary Islands, Iraq, Israel, Saudi Arabia, United Arab Emirates, Iran, India (Norrbom et al. 1999, Heřman and Dirlbek 2006, David and Ramani 2011, Gharajedaghi et al. 2012, Merz 2011).

##### Tribe TERELLINI Hendel, 1927

###### Genus *CHAETORELLIA* Hendel, 1927

####### Chaetorellia conjuncta

Taxon classificationAnimaliaDipteraTephritidae

(Becker, 1912)

######## New records.

Morocco, Anti Atlas: Airport Sidi Ifni, 5♂♂, 5♀♀, 17-V-2015; Oued Assa: 3♂♂, 2♀♀, 21-V-2015; Oued Sayad, 1♂, 25-V-2015; Oued Foum Ziguid (Douar Ouaiftoute), 1♀, 01-VI-2015; Ksibat Elhdeb, 1♂, 07-VI-2015; Oued Ziz (Pont Errachidia), 1♀, 09-VI-2015 (net sweeping)- 1♀, 12-VI-2015- 3♂♂, 23-VI-2015 (reared); Oued Ouarzazate, 4♂♂, 2♀♀, 12-VI-2015 (net sweeping)- 1♂, 28-VI-2015- 1♂, 1♀, 01-VII-2015 (reared). First record for Morocco.

######## Host plants.

Flower heads of *Centaurea calcitrapa* L. from which the specimens were reared.

######## World distribution.

Egypt, Afghanistan, Albania, Caucasus, Cyprus, Greece, Hungary, Iraq, Iran, Israel, Jordan, Kazakhstan, Lebanon, Pakistan, Syria, Turkey (White and Marquardt 1989, Norrbom et al. 1999, Korneyev and Dirlbek 2000, Merz 2000).

####### Chaetorellia hestia

Taxon classificationAnimaliaDipteraTephritidae

Hering, 1937

######## Literature record.

Morocco, High Atlas: Essaouira (Séguy 1930). First record for the Anti Atlas.

######## New record.

Morocco, Anti Atlas: Centre Sidi Ouassay, 2♂♂, 12-V-2015 (net sweeping).

######## World distribution.

Algeria, France, Spain, Italy (Séguy 1930, White and Marquardt 1989, Norrbom et al. 1999).

###### Genus *TERELLIA* Robineau-Desvoidy, 1830

####### Terellia colon

Taxon classificationAnimaliaDipteraTephritidae

Meigen, 1826

######## Literature records.

Morocco (Séguy 1930).

######## World distribution.

North Africa, Austria, Azerbaijan, Belgium, Great Britain, Crete, Czech Republic, Denmark, Dodecanese Islands, France, Germany, Georgia, Greece, Hungary, Israel, Italy, Kazakhstan, Kyrgyzstan, Lithuania, Moldova, North Aegean Islands, Poland, Romania, Russia, Sicily, Slovakia, Spain, Sweden, Switzerland, West Siberia, Tajikistan, Netherlands, Turkmenistan, Turkey, Ukraine, Uzbekistan (Foote 1984, Norrbom et al. 1999, Kütük and Özgür 2003, Özgür and Kütük 2003, Merz and Korneyev 2004, Heřman and Dirlbek 2006).

####### Terellia fuscicornis

Taxon classificationAnimaliaDipteraTephritidae

(Loew, 1844)

######## Literature records.

Morocco (Séguy 1930).

######## World distribution.

North Africa, Crete, Cyprus, France, Greece, Italy, Madeira, Malta, Sardinia, Sicily, Spain, Iran, Israel, Lebanon, Turkey, California, Scotland (Norrbom et al. 1999, Merz 2001, Whittington 2002, Kütük and Yaran 2011, Mohamadzade Namin 2011).

####### Terellia longicauda

Taxon classificationAnimaliaDipteraTephritidae

(Meigen, 1838)

######## Literature records.

Morocco, High Atlas: Tizi n’Test, Middle Atlas: Aïn Leuh (Séguy 1930, 1934b).

######## World distribution.

Albania, Austria, Great Britain, Bulgaria, Czech Republic, Cyprus, France, Germany, Hungary, Italy, Near East, Poland, Russia, Slovakia, Spain, Switzerland, Ukraine, Turkey (Kütük 2003, Merz and Korneyev 2004).

####### Terellia sp. near longicauda

Taxon classificationAnimaliaDipteraTephritidae

######## New record.

Morocco, Rif: Douar Kitane, 4♂♂, 10-VIII-2016- 2♂♂, 3♀♀, 11-VIII-2016- 1♂, 3♀♀, 12-VIII-2016- 1♀, 13-VIII-2016 (reared). First record for Morocco and North Africa.

######## Host plants.

**Figure 6.**
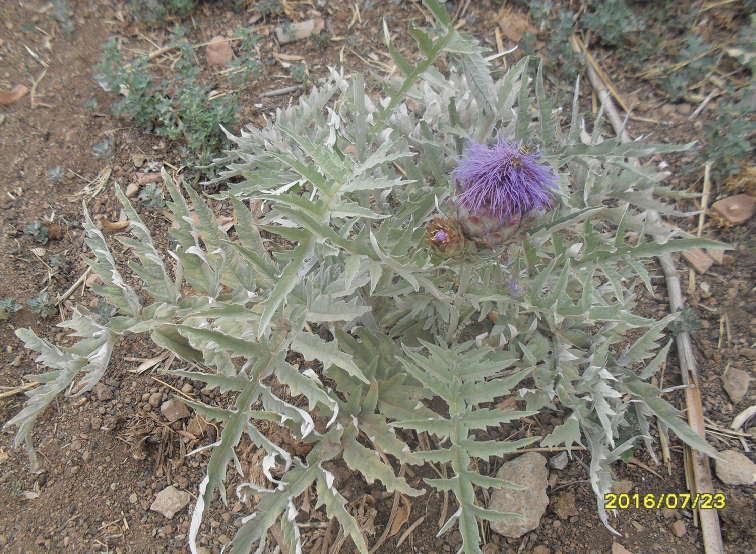
*Cynara cardunculus* L. host plant of Terellia sp. near longicauda.

Flower heads of *Cynara cardunculus* L. (Fig. 6) from which the specimens were reared.

######## World distribution.

Spain, Canary Islands (Merz 1992).

######## Comments.

This is an undescribed, cryptic species of the *Terellia serratulae* L. complex known to infest *Cynara cardunculus* L. in Spain and the Canary Islands. The *serratula* group contains cryptic species, a situation that needs to be resolved, and for which a molecular approach should be adopted (Valery Korneyev, pers. comm.).

####### Terellia serratulae

Taxon classificationAnimaliaDipteraTephritidae

(Linnaeus, 1758)

######## Literature records.

Morocco, Rif: Tangier (Wiedemann 1824, Becker and Stein 1913).

######## New sites.

Morocco, Rif: Daya Jbel Zemzem, 1♀, 23-IV-2014; Oued Maâza (Tarik El Ouasâa), 2♂♂, 1♀, 18-VI-2014 (net sweeping).

######## World distribution.

Algeria, Tunisia, Albania, Andorra, Armenia, Austria, Azerbaijan, Balearic Islands, Belgium, Great Britain, Bulgaria, China, Corsica, Crete, Cyprus, Czech Rep., Denmark, Finland, France, Georgia, Germany, Greece, Iran, Iraq, Ireland, Israel, Italy, Kazakhstan, Korea, Lebanon, Malta, Moldova, Mongolia, Netherlands, Norway, Poland, Portugal, Romania, Russia, Sardinia, Sicily, Slovakia, Spain, Sweden, Switzerland, Syria, Turkey, Ukraine (Korneyev and Dirlbek 2000, Han and Kwon 2000, Merz 2001, Heřman and Dirlbek 2006, Kütük and Yaran 2011, Khaghaninia et al. 2012).

####### Terellia virens

Taxon classificationAnimaliaDipteraTephritidae

(Loew, 1846)

######## Literature records.

Morocco, High Atlas: Jbel Ayachi (White 1989), Tizi-n-Talrhemt (Korneyev et al. 2013). First record for the Anti Atlas.

######## New record.

Morocco, Anti Atlas: Airport Sidi Ifni, 2♂♂, 17-V-2015; Oued Ouarzazate, 5♂♂, 12-VI-2015 (net sweeping)- 1♀, 01-VII-2015 (reared).

######## Host plants.

Flower heads of *Centaurea calcitrapa* L. from which the specimens were reared.

######## World distribution.

Tunisia, Albania, Afghanistan, Austria, Bulgaria, Corsica, Czech Rep., France, Germany, Greece, Hungary, Iran, Iraq, Israel, Italy, Jordan, Kazakhstan, Moldova, Netherlands, Poland, Romania, Russia, Serbia, Sicily, Slovakia, Spain, Switzerland, Turkey, Ukraine, USA (Séguy 1930, Kütük and Yaran 2011, Gharajedaghi et al. 2012, Korneyev et al. 2013).

#### Subfamily TRYPETINAE (Robineau-Desvoidy, 1830)

##### Tribe CARPOMYINI Korneyev, 1995

###### Subtribe CARPOMYINA Norrbom, 1989

####### Genus *CARPOMYA* Costa, 1854

######## Carpomya incompleta

Taxon classificationAnimaliaDipteraTephritidae

(Becker, 1903)

######### New record.

Morocco, Anti Atlas: Douar Zaouia, 1♂, 1♀, 11-VI-2015 (net sweeping). First record for Morocco.

######### World distribution.

Italy, Egypt, Ethiopia, Iraq, Israel, Saudi Arabia, Sudan, United Arab Emirates, Burkina Faso, Kenya (Norrbom et al. 1999, Merz 2011, Copeland 2009, Tankoano et al. 2012).

##### Tribe TRYPETINI Loew, 1861

###### Subtribe CHETOSTOMATINA

####### Genus *CHETOSTOMA* Rondani, 1856

######## Chetostoma curvinerve

Taxon classificationAnimaliaDipteraTephritidae

Rondani, 1856

######### New record.

Morocco, Rif: Oued El Kelâa, 1♂, 18-XII-2015; Bab El Karne, 1♀, 25-XII-2015 (net sweeping). First record for Morocco.

######### World distribution.

North Africa, Austria, Great Britain, Italy, Netherlands, Portugal, Spain, Switzerland, Uzbekistan, Iran, Israel (Norrbom et al., 1999, Mohamadzade Namin et al. 2010).

###### Subtribe TRYPETINA Loew, 1861

####### Genus *EULEIA* Walker, 1835

######## Euleia heraclei

Taxon classificationAnimaliaDipteraTephritidae

(Linnaeus, 1758)

######### Literature records.

Morocco, Gharb plain: Sidi Slimane (Séguy 1953). First records for the Rif.

######### New record.

Morocco, Rif: Oued Boumarouil, 1♂, 10-V-2014; Aïn El Âakba Larbaâ, 1♂, 18-IV-2015 (net sweeping).

######### World distribution.

Algeria, Madeira, Great Britain, Czech Rep., Estonia, Latvia, Moldova, Poland, Romania, Russia, Spain, Switzerland, Ukraine, Afghanistan, Armenia, Azerbaijan, Georgia, Iran, Israel, Kazakhstan, Kyrgyzstan, Tadjikistan, Turkey, Turkmenistan, Uzbekistan, Japan (Norrbom et al. 1999, Heřman and Dirlbek 2006, Gilasian and Merz 2008).

######## Euleia marmorea

Taxon classificationAnimaliaDipteraTephritidae

(Fabricius, 1805)

######### Literature records.

Morocco, Rif: Tangier (Séguy 1930, Zimsen 1964).

## Supplementary Material

XML Treatment for Capparimyia savastani

XML Treatment for Ceratitis capitata

XML Treatment for Bactrocera oleae

XML Treatment for Dacus frontalis

XML Treatment for Dacus longistylus

XML Treatment for Oedaspis daphnea

XML Treatment for Oedaspis multifasciata

XML Treatment for Oedaspis trotteriana

XML Treatment for Myopites inulaedyssentericae

XML Treatment for Myopites stylatus

XML Treatment for Urophora sp. near congrua

XML Treatment for Urophora mauritanica

XML Treatment for Urophora quadrifasciata

XML Treatment for Urophora solstitialis

XML Treatment for Ensina sonchi

XML Treatment for Hypenidium graecum

XML Treatment for Aciura coryli

XML Treatment for Oxyaciura tibialis

XML Treatment for Sphaeniscus filiolus

XML Treatment for Acanthiophilus helianthi

XML Treatment for Campiglossa martii

XML Treatment for Campiglossa producta

XML Treatment for Capitites ramulosa

XML Treatment for Desmella rostellata

XML Treatment for Dioxyna sororcula

XML Treatment for Euaresta bullans

XML Treatment for Goniurellia longicauda

XML Treatment for Goniurellia persignata

XML Treatment for Spathulina sicula

XML Treatment for Sphenella marginata

XML Treatment for Tephritis dioscurea

XML Treatment for Tephritis divisa

XML Treatment for Tephritis formosa

XML Treatment for Tephritis leontodontis

XML Treatment for Tephritis matricariae

XML Treatment for Tephritis nigricauda

XML Treatment for Tephritis postica

XML Treatment for Tephritis praecox

XML Treatment for Tephritis pulchra

XML Treatment for Tephritis simplex

XML Treatment for Tephritis stictica

XML Treatment for Tephritis theryi

XML Treatment for Tephritis vespertina

XML Treatment for Tephritomyia lauta

XML Treatment for Trupanea amoena

XML Treatment for Trupanea guimari

XML Treatment for Trupanea stellata

XML Treatment for Chaetorellia conjuncta

XML Treatment for Chaetorellia hestia

XML Treatment for Terellia colon

XML Treatment for Terellia fuscicornis

XML Treatment for Terellia longicauda

XML Treatment for Terellia sp. near longicauda

XML Treatment for Terellia serratulae

XML Treatment for Terellia virens

XML Treatment for Carpomya incompleta

XML Treatment for Chetostoma curvinerve

XML Treatment for Euleia heraclei

XML Treatment for Euleia marmorea
